# Supplementary material for: Iron deficiency anemia among children aged 2–5 years in southern Ethiopia: a community-based cross-sectional study
Source: PeerJ. 2021 Jun 28;9:e11649. doi: 10.7717/peerj.11649 (PMC8247708; doi:10.7717/peerj.11649)
Supplement: Supplemental Information 1 [file peerj-09-11649-s001.pdf]

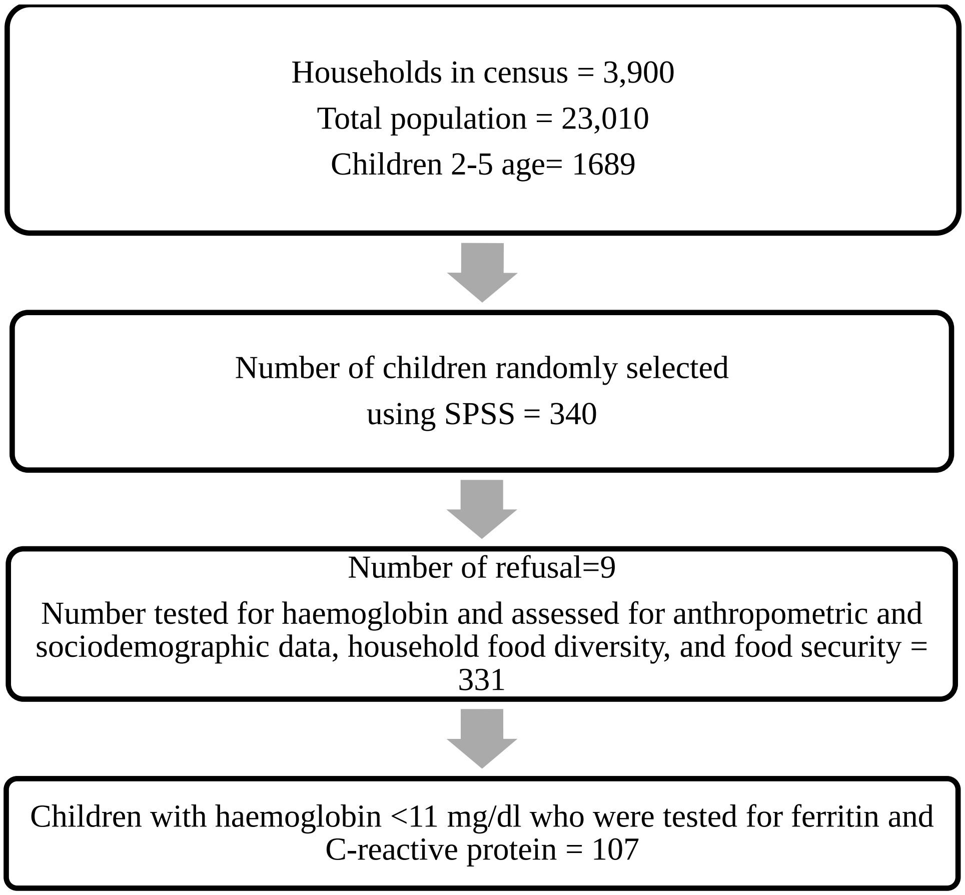
Households in census = 3,900
Total population = 23,010
Children 2-5 age= 1689
Number of children randomly selected using SPSS = 340
Number of refusal=9
Number tested for haemoglobin and assessed for anthropometric and sociodemographic data, household food diversity, and food security = 331
Children with haemoglobin <11 mg/dl who were tested for ferritin and C-reactive protein = 107
