# Supplementary figures and images for: Iron deficiency anemia among children aged 2–5 years in southern Ethiopia: a community-based cross-sectional study
Source: PeerJ. 2021 Jun 28;9:e11649. doi: 10.7717/peerj.11649 (PMC8247708; doi:10.7717/peerj.11649)

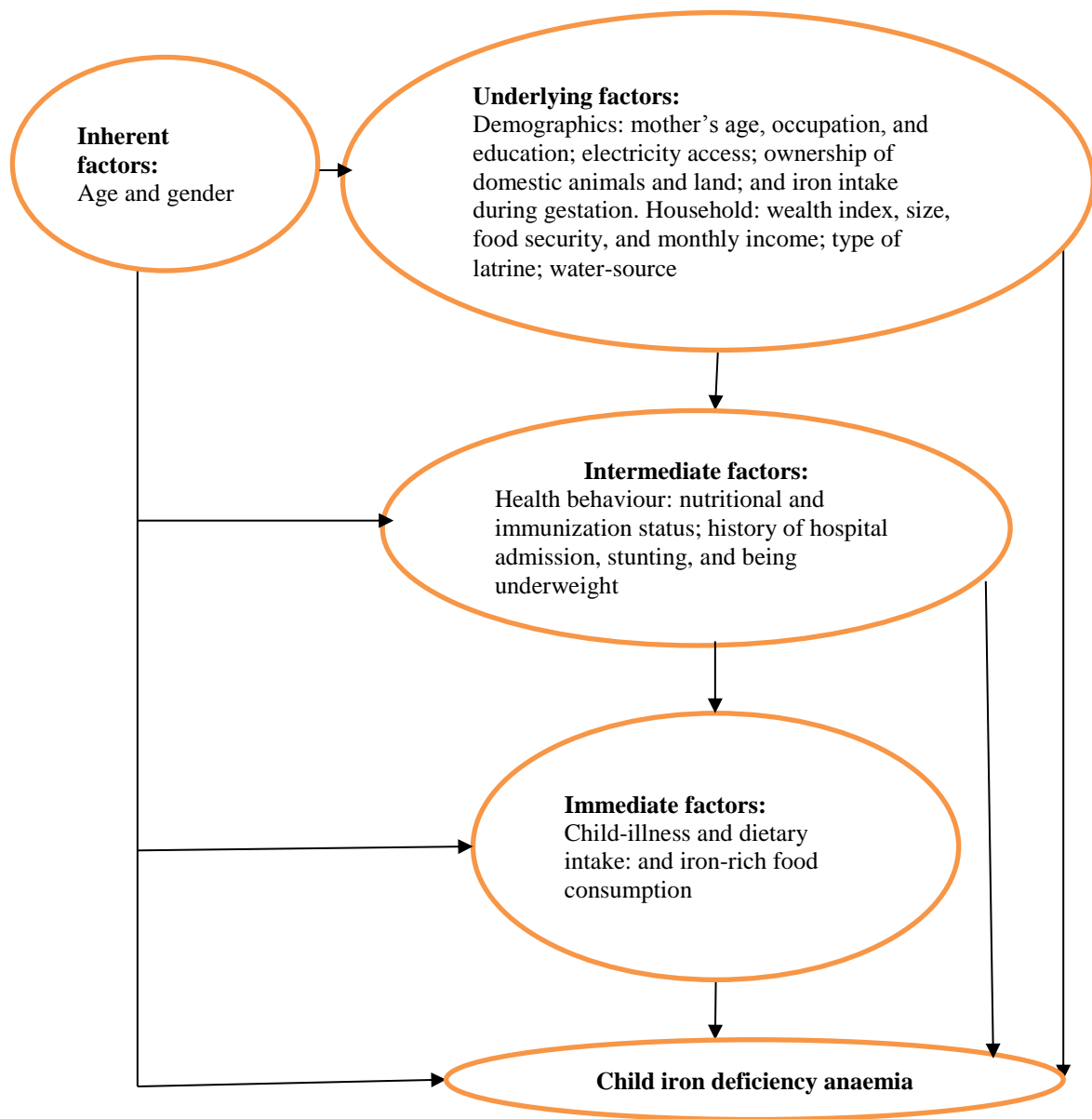

Supplement: Supplemental Information 2 [file peerj-09-11649-s002.pdf]
