# Supplementary material for: Iron deficiency anemia among children aged 2–5 years in southern Ethiopia: a community-based cross-sectional study
Source: PeerJ. 2021 Jun 28;9:e11649. doi: 10.7717/peerj.11649 (PMC8247708; doi:10.7717/peerj.11649)
Supplement: Supplemental Information 3 [file peerj-09-11649-s003.pdf]

Characteristics	Iron rich food consumption at list one time with in the last 7 days		Crude OR (95%CI)	Adjusted OR (95%CI)
	No	Yes		
Child sex				
Male	120 (52.9)	52 (50.0)	1	1
Female	107 (47.1)	52 (50.5)	0.89 (0.56-1.42)	0.93 (0.55-1.56)
Child age in month			1.01 (0.99-1.03)	1.01 (0.99-1.04)
Household dietary diversity (continuous)			1.53 (1.33-1.76)	1.44 (1.23-1.67)
Meal frequency per day (continuous)			1.57 (1.13-2.19)	1.27 (0.88-1.83)
Mother school attended years (continuous)			1.14 (1.07-1.20)	1.13 (1.03-1.24)
Father school attended years (continuous)			1.07 (1.02-1.14)	0.93 (0.85-1.02)
