# Supplementary material for: Iron deficiency anemia among children aged 2–5 years in southern Ethiopia: a community-based cross-sectional study
Source: PeerJ. 2021 Jun 28;9:e11649. doi: 10.7717/peerj.11649 (PMC8247708; doi:10.7717/peerj.11649)
Supplement: Supplemental Information 5 [file peerj-09-11649-s005.pdf]

Iron deficiency anemia among children aged 2-5 years in southern Ethiopia: a community based cross-sectional study

STROBE Statement—Checklist

	Item No	Recommendation	Page No
**Title and abstract**	1	(*a*) Indicate the study's design with a commonly used term in the title or the abstract	Title Page1
		(*b*) Provide in the abstract an informative and balanced summary of what was done and what was found	Abstract Page 2
**Introduction**			
Background/rationale	2	Explain the scientific background and rationale for the investigation being reported	Page 4
Objectives	3	State specific objectives, including any pre-specified hypotheses	Page4
**Methods**			
Study design	4	Present key elements of study design early in the paper	Page 5
Setting	5	Describe the setting, locations, and relevant dates, including periods of recruitment, exposure, follow-up, and data collection	Page 5, study area
Participants	6	(*a*) Give the eligibility criteria, and the sources and methods of selection of participants	Page 6, study participants
Variables	7	Clearly define all outcomes, exposures, predictors, potential confounders, and effect modifiers. Give diagnostic criteria, if applicable	Page 6, study variables
Data sources/ measurement	8*	For each variable of interest, give sources of data and details of methods of assessment (measurement). Describe comparability of assessment methods if there is more than one group	Page 6-10, blood collection and laboratory, anthropometry and questionnaire
Bias	9	Describe any efforts to address potential sources of bias	Page 11-12, Data quality and control
Study size	10	Explain how the study size was arrived at	Page 5-6 sample size and Fig S1
Quantitative variables	11	Explain how quantitative variables were handled in the analyses. If applicable, describe which groupings were chosen and why	Page 10-11, statistical analysis
Statistical methods	12	(*a*) Describe all statistical methods, including those used to control for confounding	Page 10-11 statistical analysis and<br>Figure2
		(*b*) Describe any methods used to examine subgroups and interactions	NA
		(*c*) Explain how missing data were addressed	NA

		(*d*) If applicable, describe analytical methods taking account of sampling strategy	NA
		(*e*) Describe any sensitivity analyses	NA
**Results**			
Participants	13*	(a) Report numbers of individuals at each stage of study—eg numbers potentially eligible, examined for eligibility, confirmed eligible, included in the study, completing follow-up, and analysed	Page 12
		(b) Give reasons for non-participation at each stage	NA
		(c) Consider use of a flow diagram	NA
Descriptive data	14*	(a) Give characteristics of study participants (eg demographic, clinical, social) and information on exposures and potential confounders	Page 12-14, Child characteristics and Socio demographic characteristics of mother and households
		(b) Indicate number of participants with missing data for each variable of interest	Page (Table 1,2 and 3)
Outcome data	15*	Report numbers of outcome events or summary measures	Page 12-13 Table 5and 6.
Main results	16	(*a*) Give unadjusted estimates and, if applicable, confounder-adjusted estimates and their precision (eg, 95% confidence interval). Make clear which confounders were adjusted for and why they were included	Page 13, (Table 6).
		(*b*) Report category boundaries when continuous variables were categorized	NA
		(*c*) If relevant, consider translating estimates of relative risk into absolute risk for a meaningful time period	NA
Other analyses	17	Report other analyses done—eg analyses of subgroups and interactions, and sensitivity analyses	NA
**Discussion**			
Key results	18	Summarise key results with reference to study objectives	Page 14, discussion
Limitations	19	Discuss limitations of the study, taking into account sources of potential bias or imprecision. Discuss both direction and magnitude of any potential bias	Page 15-16, discussion
Interpretation	20	Give a cautious overall interpretation of results considering objectives, limitations, multiplicity of analyses, results from similar studies, and other relevant evidence	Page 16
Generalisability	21	Discuss the generalisability (external validity) of the study results	Page 16, conclusion
**Other information**			
Funding	22	Give the source of funding and the role of the funders for the present study and, if applicable, for the original study on which the present article is	

based	
