# Supplementary material for: Iron deficiency anemia among children aged 2–5 years in southern Ethiopia: a community-based cross-sectional study
Source: PeerJ. 2021 Jun 28;9:e11649. doi: 10.7717/peerj.11649 (PMC8247708; doi:10.7717/peerj.11649)
Supplement: Supplemental Information 6 [file peerj-09-11649-s006.pdf]

SURVEY QUESTIONNAIRE - IRON DEFICIENCY ANEMIA AMONG CHILDREN 2-5 YEARS OLD IN ETHIOPIA

DATE OF INTERVIEW |____|____| Day |___|___| Month |__|__||__|__| Year

TIME STARTED |__ |__| Hour |__|__| Minutes

TIME ENDED |__ |__| Hour |__ |__ | Minutes

INTERVIEWER NAME ______________________________________

SUPERVISOR NAME ____

CHECKED BY ____

ENTERED BY ____

LOCALITY NAME____

VILLAGE NAME ____

NAME OF HOUSEHOLD HEAD____

NAME OF MOTHER ____

NAME OF CHILD ____

HOUSEHOLD IDENTITY NUMBER____

KEBELE CODE____

Part I

Socio demographic characteristics of the mother or care giver of the child

Ask the caregiver to find the vaccination card (cross-check all information with the card)

እናትየውን የክትባት ካርድ ጠይቀህ/ሽ የምትሰ ጠውን መልስ ከካርዱ ጋር አስተያይ/ዪ

No	Question	English	Sidamoffo	
101	**How long have you been living here as household/family? Put in year**			
	Tenne mini maate ledo megegshi yanna heerita? Diro KUlie			
	ቤተሰብህ/ሽ ለምን ያህል ጊዜ እዚህ ቆያቸሁ; በአመት አስቀምጥ/ጪ (ከ 6 ወር በታች ከሆነ =0 ፤ ከ 6 ወር በላይ ከሆነ =1 አስቀመጥ/ጭ)			
102	**Did your household planed to live here for the next one year?**			
	Danno diro konne heerate hedo noohe?	0. [_] No	0. [_] Dinoe	0. [_] የለውም
	ቤተሰብሽ ለሚቀጥለው 1 አመት እዚህ የመቆየት እቅድ አላችሁ?	1. [_] Yes	1. [_] Nooe	1. [_]አለው
103	**How old is this child? (age in month) and date of birth in GC and EC**			
	3a. Qaaqqu ilamino barra(Itiyopiyu kiironni)		____,_____,___	___,_____,___
	ህጻኑ የተወለደበት ቀን (በኢትዩጰያ አቆጣጠር)			
	3b. Illamino barra (aroyopu kiirronni)		_____,_____,___	_____,_____,___
	ህጻኑ የተወለደበት ቀን (በአውሮፓ) አቆጣጠር			
	3c. Ilamino barra afamannoki haikkiro qaaqqu diro agaanunni		_________	___________
	የተወለደበት ቀን የማይታወቅ ከሆነ የልጁ እድሜ በወር			
04	**How old are you? (mother age in years)**			
	Dirikki meeho? Kiirotenni kulie.			
	የእናትየው ዕድሜ ስንት ነው ? (በአመት)			
	**Is the child a boy or a girl?**			
105	Daaimu Koo/tee? Meyatenso labaho	1. [_] Boy	1. [_] Labaaho	[_] 1.ወንድ

	ሕፃኑ ወንድ ነው ሴት?	2. [_] Girl	2. [_] Meyaate	[_] 2.ሴት
106	**What is your relationship with the child?**			
	Daaimuua ate fixoomi maati?	1.[_] Mother	1.[_]Amate	1[_] እናት
	አንቺ ለህፃኑ ምኑ ነሽ?	2.[_] Father	2.[_]Annaho	2[_] አባት
		3.[_] Step mother /father	3.[_]Buddeenu amate	3[_] እንጀራ አናት/ባት
		4.[_] Grand mother/Father	4.[_]Ahaahete/ hoho	4[_] አያት
		5.[_] Sister/brother	5.[_]Rodoote/Rodooho	5[_]እህት/ወንድም
		6.[_] Uncle/Aunt	6.[_] Aboho/halamete	6[_] አጉት/አክስት
		7.[_] Other specify	7.[_]Welere	7[_]ሌላ/ዘርዝር
107	**What is your religion**			
	Anunoki meati?	1.[_]Orthodox	1.[_]Orthodoksete	1[_] አርቶደክስ
	ሀይማኖትሽ ምንድን ነው?	2.[_]Protestant	2.[_] Protestantete	2[_] ጴንጤ
		3.[_]Catholic	3.[_] Katoolikete	3[_] ካቶሊክ
		4.[_]Muslim	4.[_] Isilamaho	4[_] ሙስሊም
		5.[_]Traditional	5.[_] Budu amanooti	5[_] ባህላዊ
		6.[_]Other specify	6.[_] Wolere	6[_] ሌላ/ዘርዝር
108	**What is your marital status?**			
	Mine kalaqirooto/ta?	1.[_]Single	1.[_]Minaamedti	1[_] ያገባ
	የጋብቻ ሁኔታሽ/ህ ምን ይመስላል?	2. [_] Married	2.[_]Qeedhichchaho	2[_] ያለገባ
		3. [_] Divorced	3.[_] Fateherawase	3[_] ተራርቆ የሚኖር
		4. [_] Widowed	4.[_] Tiroomo/ma	4[_] የተፋታ/ች
		5. [_] Separated	5.[_]Shirroomo/ma	5[_] የሞተባት/በት
109	**How many years of completed school does the mother have?**			
	Rosu aana mee diro sayisootta			
	በትምህርት ላይ ምን ያህል አመት አሳልፈሻል?			
110	**How many years of completed school does the father have?**			

	Annu rosu mine mee dirosayisino	
	የህፃኑ አባት በትምህርት ቤት ምን ያህል አመት አሳልፏል?	
111	**What is the total number of people living in your household?**	
	Tenne mini maate eiddo moo manni kirro meeho?	
	በዚህ ቤት ውስጥ ምን ያህል ሰው ነው የሚኖረው?	
11A	**A. How many are younger than 5 year?**	
	Ontu dirii worihu meeho?	
	ከ5 አመት በታች ምን ያህል ናቸው?	
11B	**How many are 5 and older but younger than 18year?**	
	Ontu dirii alihuna 18 dirii worihu nee mannati?	
	በ5 አመትና በ18 መከከል ምን ያህል ናቸው?	
11C	**How many are 19 up to 59year?**	
	19-59diri geesha nee mannaati?	
	ከ19-59 አመት መካከል ምን ያህል ናቸው?	
11D	**How many are older than 60 year?**	
	60 diri alihu meeho?	
	ከ60 አመት በላይ ምን ያህል ናቸው?	

Part 2 Question on Economic status of the house hold

Kirro 2 Mini manni maatete jirote bikka

ክፍል 2. የቤተሰብ ምጣኔ ሀብት መለኪያ መጠይቅ

201	**What is the mother occupational status?**			
	Amote loosi hiitooho?	1.[_] Unemployed	1.[_]Loosu dinooho	1[_]ስራ የሌለው
	የእናትየው ዋና ስራ ምንድን ነው?	2. [_] Day laborer	2.[_]Barru loosasicho	2[_]የቀን ሰራተኛ
		3. [_] Farmer	3.[_] Baatote looso	3[_]ገበሬ

		4. [_] Merchant	4.[_] Dadalanchoho	4[_]ነጋዴ
		5. [_] NGO employed	5.[_]Mengistati looso	5[_]የድርጅት
		6.[_]Government employed	6.[_]Manaisete looso	6[_]የመንግሰት
		7. [_] Student	7.[_]Rosaanchoho	7[_]ተማሪ
		8.[_]Other specify	8.[_]wolere xawisi	8[_]ሌላ ካለ ግለፅ
202	**What is the Father occupational status?**			
	Annu Loosi hiittooho?	1.[_] Unemployed	1.[_]Loosu dinooho	1[_]ሰራ የሌለው
	የአባትየው የስራ ሁኔታ?	2. [_] Day laborer	2.[_]Barru loosasicho	2[_]የቀን ሰራተኛ
		3.[_]Farmer	3.[_] Baatote looso	3[_]ገበሬ
		4.[_]Merchant	4.[_] Dadalanchoho	4[_]ነጋዴ
		5.[_]NGO employed	5.[_]Mengistati looso	5[_]የድርጅት
		6.[_]Government employed	6.[_]Manaisete looso	6[_]የመንግሰት
		7.[_]Student	7.[_]Rosaanchoho	7[_]ተማሪ
		8.[_]Other specify	8.[_]wolere xawisi	8[_]ሌላ ካለ ግለፅ
203	**What was last month income?**			
	Sai aganira megeeshi afidhinoonni			
	ባለፈው ወር ቤተሰቡ ያሰገባው ወራዊ ገቢ ምን ያህል ነበር?			
	**What is the average yearly income of the household?**			
204	Dirunnit mereerima eone megeeshshaatic?			
	በአማካይ የቤተሰቡ አመታዊ ገቢ ምን ያህል ነበር?			
205	**How much do you save yearly?**			
	Dirruni megeeshshi woxe suuqisidhinenni?			
	በአመት ምን ያህል ትቆጥባላችሁ?			
206	**House hold facility : Do you have any of the following**			
	Mini meate injo konniaane nori gido kinera noori noo’ne?	1.[_]Electricity	1[_] Maabraate	1[_] ማብራት

	በቤት ውሰጥ ከሚገኙ መገልገያ ቁሶች የትኞቹ አሉሽ?(የሌላትን **0** ሙላ)	2.[_]Radio	2[_] Radoone	2[_] ራዲዖ
		3.[_]Mobile telephone	3[_]Silke	3[_] ሰልክ
		4.[_]Non mobile telephone	4[_] Teevizhiine	4[_] ቴሌቪዥን
		5.[_]Television	5[_]Qiissaasinchu	5[_] ፍሪጅ
		6.[_]Computer	6[_]Maabiraatete	6[_] የማብራት ምድጃ
		7.[_]Refrigerator	7[_] Compitere	7[_] ኮምፒውተር
		8.[_]Electric stove	8[_] Sayiikile	8[_] ሳይክል
		9.[_]Motor bike	9[_]Motorete sayikle	9[_] ሞተር ሳይክል
		10.[_]Car	10[_] መኪና	10[_] መኪና
		11.[_]Other specify	11[_] ሌላ ካለ ግለፅ	11[_] ሌላ ካለ ግለፅ
207	**Does the household own any agricultural land?**			
	Konni mini maatera umi'ne baato loosidhinenniti noo'ne?	0. [_] No	0. [_] Dinoe	0[_] የለውም
	የዚህ ቤት ባለቤት የእርሻ መሬት አለው?	1. [_] Yes	1. [_] Nooe	1[_] አዎ
208	**How many (LOCAL UNITS) of agricultural land do this household own?**			
	Allanne halaligne mepeeshite (Kine keeninni) Akine umine baattoti?			
	የእርሻ መሬት ካለው ምን ያህል መሬት ነው ያለው (በባህለዊ መለኪያ አስቀምጥ)			
209	**Does the house hold produce any yield with this land**			
	Baatone loosine guma afidhinenni?	0. [_] No	0. [_]Diafineeno	0[_] የለውም
	በዚህ መሬት የምታመርቱት ምርት አለ?	1. [_] Yes	1. [_]Afineeno	1[_] አዎ
210	**Does your house hold own any domestic animal**			
	Konni mini maatera mini saado no?	1. [_] Yes	0. [_] Dinoe	0 [_].አይደለም
	የቤት እንስሳት አላችሁ?	2. [_] No	1. [_] Nooe	1 [_].አዎ
211	**How much of the following do you have?**<br>Afidhinoonniha ikkiro hiite saada? Megessha?	Chiken	1.Lukkicho____	1. ዶሮ ____

	የቤት እንሰሳ ካለችሁ ከሚከተሉት ውስጥ ምን ያህል ዐላችሁ?**(የሌለውን 0 አስቀምጥ)**	Goat	2. Meicho_____	2. ፍየል____
ዝለል Skip		Sheep	3.Gereewo____	3. በግ ____
		Ox	4.Bootta_______	4. በሬ ________
		Cow	5.Lalo______	5. ላም ____
		Donkey	6.Harricho____	6. አህያ ____
		Other specify	7.wolere xawisi_______	7. ሌላ ዘርዝሪ/ር____
212 ዝለል Skip	**If yes for question number 211 Do you feed animal products for your children**			
	Saedate winni afidhi nannire daaimaho uyitinnan?	0.[_]No	0. [_] Dinoe	0. [_] አላበላም
	የራሰሽን የከብቶች ተዋፅኦ ለልጆችሽ ታበያለሽ?	1.[_]Yes	1. [_] Nooe	1. [_] አዎ
213	**What type of latrine do you have**			
	Shumate mini hittoohu noone?	1.[_]No facility/bush/field	1[_]Dinoe	1[_]የለንም
	ምን አይነት ሽንት ቤት ነው ያላችሁ?	2.**[_]**Composting toilet	2[_]Irshu giddo	2[_]የማሳ ውሰጥ
		3.[_]Open pit	3[_]Haqqunnabushshunni calla tu'nooni	3[_]ክፍት ጉድጎድ
		4.[_]Pit latrine with slab	4[_]Simmintoteni loonsoonnishuma mine.	4[_]የወለል ልባስ ያለው የጉድጎድ ሽንት ቤት
		5.[_]Ventilated improved pit latrine (vip)	5[_]Tuubbotenni foolanno shuamte mine.	5[_]የተሻሻለ የጉድጎድ ሽንት ቤት
		6.[_]Flush or pour flush toilet	6[_]Wiyiinni loosanno shumate mine.	6[_]በውሀ የሚሄድ ሽንት ቤት
214	**What is the main source of drinking water for your house hold**			
	Waa horonsidhinonnihu maminniti?	1.[_]unprotected well/spring	1[_]Huxxinoonnkki buichcho	1[_]ካልተከለለ የከርሰ ምድርውሀ
	የመጠጥ ውሀ በዋናነት ከየት ነው የምትጠቀሙት?	2.[_]protected spring/well	2[_]Huxxinoonni buichcho	2[_]ከተከለለ የከርሰ ምድር ውሀ
		3.[_]tanker truck	3[_]Rottote giddo kuusanino waa	3[_]ከማጠራቀሚያ
		4.[_]public tap/standpipe	4[_]Olluu horons'rano	4[_]ከህዝብ ቧንቧ

			5[_]waa Gibbete giddo	
		5.[_]piped into dwelling	5[_]Waa Gibbete giddo	5[_]ግቢ ከገባ ቧንቧ

Part 3 child feeding practice
Kiiro 3 Qaaqqu sagalate gara
ክፍል **3** የህፃኑ አመጋገብ ሁኔታ

No	Question	Answer		መልስ
301	**Do you feed breast milk to your child?**			
	Daaima unuuna qansata?	1. [_] Yes	1. [_] Qanseema	1[_] አዎ
	ህፃኑን ዐሁን ጡት ታጠቢዋለሽ?	2. [_] No	2. [_]Diqaanseema	2[_] አላጠባውም
302	**If the mom is not breastfeeding now: Did you ever breastfeed your child?**			
	Ama unuunna qansitahakkiha ikkiro Qaaqqokk unuuna qansootta	1. [_] Yes	1. [_] Qanseema	1[_] አዎ
	እናትየው ዐሁን ጡት ማታጠባ ከሆነ ልጅሽን ጡት አጥብተሽው ነበር(በፊት)	2. [_] No	2. [_]Diqaanseema	2[_] አላጠባውም
303	**What is the number of meals normally taken by the child per day?**			
	Daaaimikki barrunni meu dani sagale itanno?\			
	ህፃኑ አሁን በቀን ምንያህል ጊዜ ነው የሚመገበው			
304	**Does child can feed himself/herself**			
	Daaimu sagale umisinni saga'la dandaanno?	1[_]Care giver	1[_]Manncholaatisannohu	1[_]ተንከባካቢ ያበላዋል
	ህፃኑ በራሱ መመገብ ይችላል?	2[_]With assistance	2[_]Irkotenniteitanohu	2[_]በዕገዛ ይበላል
		3[_]Self feeding	3[_]Umosit itannohu	3[_]ህፃኑ በራሱ ይበላል

Part 4: 24 Hour Dietary Diversity Questioner
Gaamo 4: 24 Sagalete danixamo
ክፍል:4:በ24 ሰአት ዉስጥ የወሰደውን የምግብ አይነት የሚገልጥ ጥያቄ

No	Type of food				

401	**I am going to ask you questions about what you fed your baby from the time you woke up yesterday morning till you woke up this morning either separately or combined with other foods.**				
	Bero soodo qoxootto wiinni kaitto yannanni hanaffe techo soodo geeshsha aante noo segalla giddo daaimaho itisootto sagale no? Itisoottaha ikkiro me’e higge itisoottoro kulattoe? Itisootto segale no<br>ዐሁን ህፃኑ ከትላነት ጠዋት ዕስከ ዛሬ ጠዋት የተመገበውን የምግብ ዐይነት ዕጠይቅሻለሁ (ህፃኑ የተመገበውን ቁርስ ፣ምሳ ዕራት ዕንዲሁም በየመሀል የተመገበውን በመጠየቅ የሚስማማውን ቦታ ጥቀስ)				
402	**Did your child eat any porridge or gruel (from what it made)**				
	Daaimu sherko woy axmiite saga’lino (mayinni qixxeessinoonniha)?	0[_] No	0.[__]Disaga’lino	0[_] አልተመ ገበም	
	ልጅሽ ከማንኛውም የእህል ዘር የተሰራ ገንፎ ወይም ሙቅ ተመግቧል?	1[_] Yes	1.[__]Saga’lino	1[_] ተመግ ቧል	
403	**Bread, pasta, rice, noodles, biscuits, cookies or any other food made from ,oats, maize, barley, wheat, sorghum millet, or other grain? Specify**				
	Daabbo,paarta,ruuze,koshoro raino sagale woy ajjunni ,badalatenni ,hayixunni,qamadetenni,bashanqunniy loonsoonni sagale woy wolu quminni,xawisi	0[_] No	0.[__]Disaga’lino	0[_] አልተመ ገበም	
	ዳቦ፤ ፓስታ፤ ሩዝ፤ ብስኩት፤ ኩኪስ፤ ወይም ማንኛውም ነገር ከአጃ ከበቆሎ ገብስ፤ ሰንዴ፤ ማሽላ፤ወይም ሌላ አህል ዘር የተሰራ	1[_] Yes	1.[__]Saga’lino	1[_] ተመግ ቧል	
404	**Any food made from teff , like injera ,kita or porridge ?**				
	Gaashetenni qqxxeessinoonnic segale buddeena, tima,woy sherko lawinore saga’lino?	0[_] No	0.[__]Disaga’lino	0[_] አልተመ ገበም	
	ማንኛውም ምግብ ከጤፍ የተሰራ (እንጀራ፤ ቂጣ፤ ገንፎ)	1[_] Yes	1.[__]Saga’lino	1[_] ተመግ ቧል	
405	**Any white potatoes, white yam?**				
	Maxaaxeesla, diinicha, boyina,lawinore saga’lino?	0[_] No	0.[__]Disaga’lino	0[_] አልተመ ገበም	

	ማንኛውም ነጭ ድንች፤ ቦይና፤ እንሰት (ማንኛውም ነጭ ስራስር)	1[_] Yes	1.[__]Saga’lino	1[_] ተመግ ቧል	
406	**Any foods made from beans, peas, lentils or pulses**				
	Aye segale baqeluuni, atarunni , qibaatete qumma shumburunni qixxeessinoonni sagale saga’lino?	0[_] No	0.[__]Disaga’lino	0[_] አልተመ ገበም	
	ባቂላ፤አተር፤ ምስር ወይም ሌላ ጥራጥሬ	1[_] Yes	1.[__]Saga’lino	1[_] ተመግ ቧል	
407	**Any nuts or seed such as peanut, sesame or sun flower seeds?**				
	Ayee qumma ocholoone coommadda gumma saga’lino	0[_] No	0.[__]Disaga’lino	0[_] አልተመ ገበም	
	ኦቾሎኒ (ከኦቾሎኒ የተሰራ ማንኛውም ምግብ)	1[_] Yes	1.[__]Saga’lino	1[_] ተመግ ቧል	
408	**Any butter,oil**				
	Zayitetenni woy buurumi loonsoonni sagala	0[_] No	0.[__]Disaga’lino	0[_] አልተመ ገበም	
	በዘይት ወይም በ	1[_] Yes	1.[__]Saga’lino	1[_] ተመግ ቧል	
409	**Any dark green, leafy vegetables like kale, spinach or amaranth leaves?**				
	Haanjarino, daraame ataakiltete daronna xu’naayye lawinore saga’lino?	0[_] No	0.[__]Disaga’lino	0[_] አልተመ ገበም	
	ማንኛውም ጥቁር አረንጎዴ አታክልት፤ ቅጠላማ አታክልት እንደ ጎመን፤ ራፎ ወይም ሌላ (ዘርዝር)	1[_] Yes	1.[__]Saga’lino	1[_] ተመግ ቧል	
	**Any pumpkin ,carrot, squash or sweet potatoes that are yellow or orange inside**				
410	Giddo bica woy haanjirino baaqulaa, kaaroote woy maxaaxeesha saga’lino?	0[_]No	0.[__]Disaga’lino	0[_] አልተመ ገበም	
	ማንኛውም ዱባ፤ ካሮት ቢጫ ሰኳር ድንች ሌላ (ዘርዝር)	1[_]Yes	1.[__]Saga’lino	1[_] ተመግ ቧል	
411	**Any ripe mangoes, papayas?**				
	Le’ado mango woy paapaayya saga’lino?	0[_]No	0.[__]Disaga’lino	0[_] አልተመ ገበም	
	የበሰለ ማንጎና ፓፓያ	1[_]Yes	1.[__]Saga’lino	1[_] ተመግ ቧል	
	**Any other fruit or vegetables**				

412	Wole aye gumma woy akaakilte saga'lino?	0[_]No	0.[__]Disaga'lino	0[_] አልተመ ገበም	
	ሌላ ማንኛውም አታክልትና ፍራፍሬ	1[_]Yes	1.[__]Saga'lino	1[_] ተመግ ቧል	
413	**Commercially fortified foods.**				
	Ashshagantinota daaimu segale?	0[_] No	0.[__]Disaga'lino	0[_] አልተመ ገበም	
	በምግብ በልፅገው የሚሸጡ ምግቦች	1[_] Yes	1.[__]Saga'lino	1[_] ተመግ ቧል	
414	**Any cheese or yogurt?**				
	Ayibe/geinto saga'lino?	0[_] No	0.[__]Disaga'lino	0[_] አልተመ ገበም	
	አይብና እርጎ	1[_] Yes	1.[__]Saga'lino	1[_] ተመግ ቧል	
415	**Frresh milk**				
	Iibbado ado	0[_] No	0.[__]Disaga'lino	0[_] አልተመ ገበም	
	ትኩስ ወተት	1[_] Yes	1.[__]Saga'lino	1 [_] ተመግ ቧል	
416	**Any eggs?**				
	Ayee quuphe	0[_] No	0.[__]Disaga'lino	0[_] አልተመ ገበም	
	እንቁላል	1[_] Yes	1.[__]Saga'lino	1[_] ተመግ ቧል	
417	**Any liver ,kidney, heart or other organ meats**				
	Afale,mule, wodana,woy wole godowu giddo maalla saga'lino?	0[_] No	0.[__]Disaga'lino	0[_] አልተመ ገበም	
	ጉበት፤ ኩለሊት፤ ልብ ወይም የእንሰሳ የውሰጥ ሰውነት ክፍል	1[_] Yes	1.[__]Saga'lino	1 [_] ተመግ ቧል	
418	**Any beef, pork, lamb, goat, rabbit (wild game meat such as antelope or deer)?**				
	Bootu maala, mancheemete maala, gereewo,mellenna hilleessa (wole dubbu saada,goljanna,guru'me lawinore segalino?	0[_] No	0.[__]Disaga'lino	0[_] አልተመ ገበም	
	የበሬ ስጋ፤ የበግ/የፍየል ስጋ የአሳማ ስጋ የጥንቸል ወይም ሌላ እንሰሳ	1[_] Yes	1.[__]Saga'lino	1[_] ተመግ ቧል	
419	**Any chicken ,duck or other birds**				
	Lukko, daakiyye, woy wole cea maala saga'lino	0[_] No	0.[__]Disaga'lino	0[_] አልተመ ገበም	
	ዶሮ፤ እርግብ ወይም የወፍ ስጋ	1[_] Yes	1.[__]Saga'lino	1[_] ተመግ ቧል	
	**Any fish**				

420	Ayee qilxi’me	0[_] No	0.[__]Disaga’lino	0[_] አልተመ ገበም	
	ማንኛውም አሳና የአሳ ምርት	1[_] Yes	1.[__]Saga’lino	1[_] ተመገ ቧል	
421	**Any soft drink specify**				
	Ayee shota agatto agino? Aginoha ikkiro xawisi	0[_] No	0.[__]Disaga’lino	0[_] አልተመ ገበም	
	ማንኛውም ለስላሳ መጠጦች	1[_] Yes	1.[__]Saga’lino	1[_] ተመገ ቧል	
422	**Coffee and Tea**				
	Buna woy shae agino	0[_] No	0.[__]Disaga’lino	0[_] አልተመ ገበም	
	ቡናና ሻይ	1[_] Yes	1.[__]Saga’lino	1[_] ተመገ ቧል	
423	Alcoholic drink				
	Diribisanno ago (birra,xesiixella,areqe)	0[_] No	0.[__]Disaga’lino	0[_] አልተመ ገበም	
	አልኮል መጠጦች (ቢራ፤ አረቄ፤ ጠጅ፤ጠላ)	1[_] Yes	1.[__]Saga’lino	1[_] ተመገ ቧል	
424	**Other specify**				
	Welere				
	ሌላ ካለ ጥቀስ				

Part 5: 7-Days Food frequency questionnaire for iron rich foods
Sufetto 5: 7 barri giddo ayirenetenni lattino sagale marri marro
ክፍል 5፤በ7ት ቀን ውስጥ ህጻኑ የወሰደው የምግብ አይነት

		**Answer the number of the day you eat**
**No**	**Question**	**መልሱን በቀናት ቁጥር ዐስቀምጥ**
501	**Now I am going to ask you if you gave the following items at all the last week ending yesterday morning. Please answer yes if you gave it and no if you did not give it And if you did, will you please tell how many times you gave it**	
	Xa xa’meemohehu sa’u lamala jeefonni kayise be’ro soodo geeshsha saga’lino sagaleeti.itinno sagale “saga’lino” itinnoki sagale ’’di’saga’lino’’ yite me’e marro itinoro xawasi.	

	እሁን የምጠይቅሽ ልጅሽ ባለፉት 7ቀን ውስጥ እስከ ዛሬ ጠዋት የበላውን የምግብ አይነት ነው ለእያንዳንዱ መልሰ ቁጥሩን ግለጪ ካልተመገበ ዜሮ መፃፍን አትርሳ(ሺ)	
502	**Food made from false banana (Kocho,kita,bula,omolicho,genfo)**	
	Inset?	
	እንሰት(ቆጮ፣ ቡላ...)?	
503	**Cereal group (maize, barley, wheat, oats, ….)**	
	Weese (badala,hayiixe	
	የእህልዘር(በቆሎ፤ ገብስ፤ ስንዴ፤ አጃ፤ ዳጉሳ፤	
504	**Pulse group (bean,pea,chickpea,….)**	
	Qixxeessinoonni (baqeluuni, atarunni ,qibaatete qumma shumburunni?)	
	የጥራጥሬ ዘር(አተር፤ ሽንብራ፤ ባቄላ፤ምስር……)	
505	**Teff (ingera,bred,porrage)**	
	Gaashe	
	ጤፍ	
506	**Peanut**	
	Ocholoone	
	ኦቾሎኒ	
507	**Dark green vegitables (Kale,green paper,qosta)**	
	Haanja daro ataakilte shaana, qaariya,Xu'naaye, raafote daro	
	ጥቁር አረንጎዴ አታክልቶች(ጎመን፤ ቃሪያ፤ ጥቁር ጎመን፤ ራፎ ……..)	
508	**Tomato, Carrot, watermelon, pumpkin**	
	Timaatime,karoote, Baaqula	
	ቲማቲም፣ ካሮት፣ ዱባ	
509	**ripe mango, papaya**	
	Lino mango,Lino pappaayya	
	የበሰለ ማንጎ፤ የበሰለ ፓፓያ	
510	**orange, lemmon,**	
	Burtukanenna loome	

	ብርቱካንና ሎሚ	
511	**Amarnth leaf or grain**	
	Raffote daronna guma	
	የራፎ ቅጠል ወይም ፍሬ	
512	**Milk and milk product**	
	Ado geinto burbuxxo	
	ወተት፤ እርጎ፤ አይብ	
513	**Any food contains butter or oil**	
	Zayitetenni woy buurumi loonsoonni sagala	
	በዘይት ወይም በቅቤ የተሰራ ምግብ	
514	**Egg**	
	Quuphphe	
	እንቁላል	
515	**Red meet (sheep, goat, ox**)	
	Dummo maala (bootunniha meichchunniha, gerechch unniha)	
	ቀይ ስጋ (የበሬ፤ የፍየል፤ የበግ)	
516	**Chicken meet**	
	Lukkichchu maala	
	የዶሮ ሰጋ	
517	**Fish**	
	Qulxume	
	አሳ	
518	**Internal organ (liver, kidney, heart)**	
	Giddoodi mannimma gaamo maalal fale(kulalitite, wodana)	
	የውስጥ የሰውነት ክፍል ሥጋ (ጉበት፣ ኩላሊት፣ልብ)	
519	**Coffe or tea**	
	Shaenna buna	
	ሻይ ወይም ቡና	
520	**Soft drink**	

	Shaffado ago	
	ለስላሳ መጠጦች	
521	**Any alchol**	
	Kajjado ago	
	ዐልኮል መጠጦች	
522	**Others specify**	
	Wole kunni assi	
	ሌላ ካለ ግለፅ/ጪ	

Part VI Supplementation question

Kifile 6 Ledishu sagale xa’mo

ክፍል 6፤ ተጨማሪ ምግብ ስለመውሰድ የሚገልጽ መጠይቅ

	**Question**			**Code**
601	**Do you feed the child any foods made with oil, fat or butter?**			
	Zayite, buuronna,coomu sagale saga’lano?	0. [_] No	0.Disagalino[__]	0[__]አይደለም
	ልጅሽን ዘይት፣ ጮማ ወይም ቅቤ ያለበትን ምግብ ትመግቢዋለሽ	1. [_] Yes	1.Sagalino[__]	1[__]አዎን
	**If yes for question number 601 do you feed every day**			
602	Barrunni daaimu me’e marro saga’lano	0. [_] No	0.Disagalino[__]	0[__]አይደለም
ዝለል Skip	ከመገብሽው በየቀኑ ትመግቢዋለሽ	1. [_] Yes	1. Sagalino[__]	1 [__]አዎን
603	**Has your child ever received Iron supplementation**			
	Daaimu ayirenete lattinota ledishu sagale afiranno?	0. [_] No	0. Disagalino[__]	0[__]አይደለም
	የደም መሙያ አንክብል ወሰዶ ያውቃል?		1. Sagalino[__]	1[__]አዎን
	**If yes for question number 603 Why he or she received?**			

604	19kki xamo saga’lanoha ikiro Mayirra sagalanno?			
ዝለል skip	የደም መሙያ አንክብል ከወሰደ ለምንድን ነው የወሰደው			
	**From where do you get?**			
605	Maminni afidhinonni?	1.[_] From health institution	1[_]Fayyimateuurishinni	1 [_]ከጤና ተቋም
ዝለል skip	የደም መሙያ አንክብል ከየት ነው የተሰጠው?	2.[_] Food aid program	2[_]Sagallte qixawonni	2 [_]ከምግብ ኘሮግራም
		3.[_]Pharmacy	3[_]Farmasetenni	3 [_]ፋርማሲ
		4. [_]Other specify	4[_]Wolekuli	4 [_]ሌላጥቀስ
606 ዝለል Skip	**Does the child taking now?**			
	Qaaqqu xaano mundee abbitawoota xagicho adhayno?	0. [_] No	0.Disagalino[__]	0[__]አይወስድም
	ህፃኑ አሁንም የደም መሙያ እንክብል አየወሰደ ነው?	1. [_] Yes	1.Sagalino[__]	1[__]አዎ
607 ዝለል Skip	**For how long he /she take supplementation?**			
	Ladishshu sagale mageshi geeeshsha sagalonno?			
	የደም መሙያ ዕንክብል ከወሰደ ለምን ያህል ጊዜ ወሰደ በወራት ጥቀሽ/ስ?			
608	**Has your child ever eat iron fortified food**			
	Daaimikki Ayiirenetenni kaajjinshoonni sagle sagalanno?	0. [_] No	0. Disagalino[__]	0[__]አይወስድም
	ህፃንሽ ደምን በሚሞላ የብረት ንጥረ ነገር የበለፀገ ምግብ በልቶ ያውቃል?	1. [_] Yes	1. Sagalino[__]	1[__]አዎ
609 ዝለል Skip	**If yes for question number 608**			
	**From where do you get**			
	Hiikiinni afiranno?	1.[_]From health institution	1[_]Fayyimateuurishinni	1[_]ጤናተቋም
	ከበላ ከየት ነው ያገኘሽው?	2.[_]Food aid program	2[_]Sagallte qixawonni	2[_]የምግብ ኘሮ ግራም
		3.[_]Pharmacy	3[_]Farmasetenni	3[_]ፋርማሲ
		4.[_]Other specify	4[_]Wolekuli	5[_]ሌላ ካለ (ዘርዝር/ሪ)

610<br>ዝለል<br>Skip	**Is the child taking now?**			
	Mamoote saga’lanno?	0.[_]No	0[_]Disagalino	0[_]አይደለም
	አሁንም ህጻኑ አየተመገበ ነው(ዕየተመገበ ከሆነ ዐሳዪኝ)?	1.[_]Yes	1[_]Sagalino	1[_]አዎን
	**What was the food**?			
611<br>ዝለል<br>Skip	Sagale maati?			
	የሚወስደው የበለጸገ ምግብ ምን ነበር(ያሳየችህን የምግብ ዐይነት ፃፍ?)			
612<br>ዝለል<br>Skip	**For how long she/he take?**			
	Mageeshshi geeshe saga’lano?			
	የበለጸገ ምግቡን ለምን ያህል ጊዜ ወሰደ(ወሰደች) በወራት ጥቀሽ/ስ?			
613	**Has the child ever been given a Vit A capsule?**			
	Daaimu vitaamine A kinine afire egennino?	0. [_] No	0. [_]Disagalino	0. [_]አያውቅም
	ህፃኑ የቫይታሚን A እንክብል ወሰዶ ያውቃል?	1. [_] Yes	1. [_]Sagalino	1. [_] አዎ
614<br>ዝለል<br>Skip	**If yes for question no 613 how often did he/she get**			
	kki xa’mo afirinnoha ikkiro ,me’e marro afirino?			
	ቫይታሚን A ከወሰደ በአመት ምን ያህል ግዜ ያገኛል?			
615	**Since you were pregnant Have you taken any Iron supplements?**			
	Godowii heedhe ayiirenete lattino sagale afiroota?	0. [_] No	0. [_]Disagalino	0. [_]አያውቅም
	እርጉዝ ሆነሽ ደም የሚሞላ እንክብል ወስደሽ ታውቂያለሽ?	1. [_] Yes	1. [_]Sagalino	1. [_] አዎ
616<br>ዝለል<br>Skip	**If yes for question number 21 for how long did you taken the supplement**			
	21kki xa’mo afirootana ikkiro mageeshi geeshsha afiroota?	1. [_] less than month	1. [_] 1 agani woro	1 [_] ከአንድ ወር በታች
	ደም የሚሞላ ዕንክብል ከወሰድሽ ለምን ያህል ጊዜ ነው የወሰድሽው?	2. [_] 1 up 2 month	2. [_] 1-2 agani geesha	2. [_] 1-2ወር

		3. [_] 2up3	3. [_] 2-3 agani geesha	3. [_] ከ2-3 ወር
		4. [_] more than 3 month	4. [_] 3 apani ale	4. [_] ከ3 ወር በላይ
		5. [_] for 6 month<br>6. [_] Do not know	5. [_] 6 adani geesha	5. [_]ለ6ወር
			6. [_] Diafooma	6. [_]ዐላውቅም

Part 7. Baby health question

Kifile 7 Daaimu fayimu xa'mu

ክፍል 7 የህፃኑን ጤና የሚገልፅ መጠይቅ

Now I am going to ask you questions which are related to your baby's health

Xa xa'meemohehu daa'mu fayima ledo xedooshu noo xa'mooti

ዐሁን የህጻኑን ዐጠቃላይ የጤና ሑኔታ የሚገልጽ ጥያቄ ዕጠይቅሻለሁ

	QUESTION		CODE	
**701**	**Does the child vaccinated ?**			
	Daaimu kittibaate garunni gudino?	0. [_] No	0. [_]Dee'ni	0. [_]አይ
	ህፃኑ ክትባት ወስዷል?	1. [_] Yes	1. [_]Eewa	1. [_]አዎ
**702**	**Does the child had vaccine card?**			
	Kittibaate gudino kaarde noosi?	0. [_] No	0. [_]Dee'ni	0. [_]አይ
	ህፃኑ የክትባት ካርድ አለው?	1. [_] Yes	1. [_]Eewa	1. [_]አዎ
	**Could you show me vaccine card (please find which vaccine the child take from the card?)**			
705	BCG kitibaate dasirino anga lea hasireemona lao?	[_]BCG?	[_]BCG?	[_]የሳንባ ምች

	Dasirinohu qinniticho angara dasahootic?	[_]Polio 0?	[_]Polio 0?	[_]የህፃናት ልምሻ 0
		[_]Polio1?	[_]Polio1?	[_]የህፃናት ልምሻ 1
	የክትባቱን ካ ርድ ታሳዩኛለሽ (በክትባት ካርድ ላይ የወሰደውን X ያልወሰደውን 0 አድርግ/ጊ)	[_]Polio2?	[_]Polio2?	[_]የህፃናት ልምሻ 2
		[_]Polio3?	[_]Polio3?	[_]የህፃናት ልምሻ 3
		[_]DPT- 1	[_]DPT- 1	[_]ዲፒቲ 1
		[_]DPT- 2	[_]DPT- 2	[_]ዲፒቲ 2
		[_]DPT- 3	[_]DPT- 3	[_]ዲፒቲ 3
		[_]PCV1	[_]PCV1	[_]ፒሲ ቪ1
		[_]PCV2?	[_]PCV2?	[_]ፒሲ ቪ 2
		[_]PCV3?	[_]PCV3?	[_]ፒሲቪ3
		[_]Rota1?	[_]Rota1?	[_]ሮታ 1
		[_]Rota2?	[_]Rota2?	[_]ሮታ 2
		[_]Measealse	[_]Measealse	[_]ኩፍኝ
703	**Can I see the BCG scar please?**			
	BCG kitibaate dasirino anga lea hasireemona lao?	1.[_]BCG lesion seen:	1.[_]BCG-bassa leellanno	1 የክትባቱ ጠባሳ ይታያል
	Dasirinohu qinniticho angara dasahootic	2.[_]BCG lesion not seen	2.[_]BCG-bassu dileellanno	2 የክትባቱ ጠባሳ አይታይም
	የሳንባ ነቀርሳ ክትባት ቦታውን ማየት እችላለው?			

Part 7.2 Diarrhea,

Diarrhea episode two weeks recall

ክፍል 7.1: ባለፉት 2 ሳምንታት ስለ ህፃኑ የተቅማጥ ህመም መጠይቅ

	QUESTION		Code	

707	**During the last two weeks that ended yesterday morning, did the child have diarrhea?**			
	Sau leme lamalanni henafe bero soodo geeshsha deuu malaati noosi?	0. [_] No	0. [_]Dinosi	0. [_]አይደለም
	ባለፉት 2 ሳምንታት ህፃኑ የተቅማጥ (የሆድ) በሽታ አሞት ነበር?	1. [_] Yes ↓	1. [_]Noosi	1. [_]አዎ
708 ዝለል Skip	**Did the child pass any watery stools?**			
	Waa lawanno deiinoosi?	0. [_] No	0. [_]Dinosi	0. [_]አይደለም
	ህፃኑ ውሀ የሚመስል ተቅምጥ አሰቀምጦታል	1. [_] Yes↓	1. [_]Noosi	1. [_]አዎ

Part 7.3: Pneumonia 2 Week recall
Kifille 7.3: Lemala mereero saambu michche xisso
ክፍል 7.3 ላለፉት 2 ሳምንታት የሳንባ ምች ህመምን የሚያመላክት መጠይቅ

	**QUESTION**		**CODE**	
**724**	**During the last two weeks that ended yesterday morning, did the child have cough?**			
	Sau lame lemale kayisse be’ro soodo geeshsha, Daaimu Buusano Afirino?	0. [_]No	0. [_]Dinosi	0. [_]አይደለም
	ባለፉት 2 ሳምንታት ህፃኑ ሳል አሞት ነበር ?	1. [_]Yes	1. [_]Noosi	1. [_]አዎን
	**During the last two weeks that ended yesterday morning, did the child have fast or difficult breathing?**			
725 ዝለል Skip	Sau lemalaa kayiise be’ro soodo geeshsha. Daaimu rahe rare fo’lanno woy foo’late rakkatanno?	0. [_]No	0. [_]Dinosi	0. [_]አይደለም
	ላለፋት 2 ሳምንታት እስከ ትላንት ጠዋት ህፃኑ ትንፋሽ ያጥረው ነበር ?	1. [_]Yes	1. [_]Noosi	1. [_]አዎን
726	**Did the illness interfere with the child ability to drink or eat?**			

ዝለል<br>Skip	Xisso ittennona agannoki gede rakkatisannosi?	0. [_]No	0. [_]Dinosi	0. [_]አይደለም
	የህመሙ ሁኔታ መብላትና መጠጣት ዕንዲያስቸግረው ዐድርጎበት ነበር?	1. [_]Yes	1. [_]Noosi	1. [_]አዎን

Part 7.4:Hospitalizations

Kiffile 7.4:Hospiitaalete goxinno yanna

ክፍል 7.4፡የህፃኑ ሆስፒታል የመተኛት ሁኔታ

	QUESTION			CODE
**729**	**Since birth has ever been admitted to hospital?**			
	Daaimu ilami yanaa henafe hospiitalete goxe egennino	0. [_] No	0. [_]Dinosi	0. [_]አይደለም
	ህፃኑ ከተወለደ ጀምሮ ሆስፒታል ተኝቶ ያውቃል?	1. [_] Yes ↓	1. [_]Noosi	1. [_]አዎን

Part 9**: Household Food Insecurity Access Scale (HFIAS) Measurement:**

Now I would like to ask you few questions regarding your household food security situation in the past four weeks.

ክፍል 9 ላለፉት 4 ሳምንታት የቤተሰቡን የምግብ ዋስትና የሚያሳይ መጠይቅ

**Rule**: We, will not give "timing" instructions and force answers.<br>Reported frequency in correct category according to this list:<br>0. No/never,<br>1. Sometimes: 1-2 days last month,<br>2. Often: 3-10 days/month,<br>3. Very often/usually: More than 10 days last month		በሚከተለው ገለፃ መሰረት የዕናትየውን መልስ ዐስቀምጥ/ጪ<br>☐1ወ ይም 2 ጊዜ ላለፉት 4 ሳምንታት (ጥቂት ጊዜ)<br>☐ከ3 -10 ጊዜ ላለፉት 4 ሳምንታት (ዐንዳንድ ጊዜ)<br>☐ከ10 ጊዜ በላይ ላለፉት 4 ሳምንታት (ብዙ ጊዜ)		
**ተ/ቁ**	**ጥያቄ (questions)**			
**901**	**In the past four weeks, did you worry that your household would not have enough food?**			
	Sau shoole lamalara miniki manni ikkado sagala afirewokkhura qarranten egenootta	0. [_]No	0. [_]Dee'ni	0. [_]አይደለም

	ባለፉት ዐራት ሳምንታት ለቤተሰብሽ በቂ ምግብ ካለመኖሩ የተነሣ ተጨንቀሽ ታውቂያለሽ	1. [_]Yes	1. [_]Eewa	1. [_]አዎን
	**How often did this happen in the past four weeks?**			
902 skip ዝለል	Qarrante egendottara shoole lamala giddo mageeshshi yanna ikkanno	[_]1.Rarely (once or twice in the pastfour weeks)	[_]1.Harancho hanna	[_]1.ጥቂት ጊዜ
	ባለፉት 4 ሳምንታት ለምን ያህል ጊዜ ዐጋጠመሽ	[_]2.Sometimes (three to ten times inthe past four weeks)	[_]2.Sae sae	[_]2. ዐንዳንድ ጊዜ
		[_]3.Often (more than ten times the past four weeks)	[_]3.Seeda yanna	[_]3. ብዙ ጊዜ
	**In the past four weeks, were you or any household member not able to eat the kinds of foods you preferred because of a lack of resources?**			
903	Sau shoole lamalara hidha hoogate kainohunni ati woy minikki maate sagala hasidhinoonni sagalete dano ita hooggine egentinoonni	0. [_]No	0. [_]Dee'ni	0. [_]አይደለም
	ባለፉት ዐራት ሳምንታት መግዛት ካለመቻል የተነሳ ዐንቺ ወይም ከቤተሰብሽ ዐባላት መብላት የፈለጋችሁትን የምግብ ዐይነት ሳትመገቡ ቀርታችሁ ታውቃላችሁ	1. [_]Yes	1. [_]Eewa	1. [_]አዎን
904 Skip ዝለል	How often did this happen in the past four weeks?			
	Hoogginihunni mageeshshi barra ikkanno xaadenehu	[_]1.Rarely (once or twice in the pastfour weeks)	[_]1.Harancho hanna	[_]1.ጥቂት ጊዜ
	ባለፉት 4 ሳምንታት ለምን ያህል ጊዜ ዐጋጠመሽ	[_]2.Sometimes (three to ten times inthe past four weeks)	[_]2.Sae sae	[_]2. ዐንዳንድ ጊዜ
		[_]3.Often (more than ten times the past four weeks)	[_]3.Seeda	[_]3. ብዙ ጊዜ
	**In the past four weeks, did you or any household member have to eat a limited variety of foods due to a lack of resources?**			
905	Sau shoole lamala hoongunni kainohunni at way minikki maate mitte bikka callo sagalete dana sagalitinoonni	0. [_]No	0. [_]Dee'ni	0. [_]አይደለም

	ባለፉት ዐራት ሳምንታት መግዛት ካለመቻል የተነሳ ዐንቺ ወይም ከቤተሰብሽ ዐባላት ውስጥ ዐንድ ዐይነት ምግብ ብቻ ተመግባችሁ ታውቃላችሁ	1. [_]Yes	1. [_]Eewa	1. [_]አዎን
906 Skip ዝለል	**How often did this happen in the past four weeks?**			
	Ikkiro sau shoole lamalara mageeshshi geeshshooti	[_]1.Rarely (once or twice in the pastfour weeks)	[_]1.Harancho hanna	[_]1.ጥቂት ጊዜ
	ባለፉት 4 ሳምንታት ለምን ያህል ጊዜ ዐጋጠመሽ	[_]2.Sometimes (three to ten times inthe past four weeks)	[_]2.Sae sae	[_]2. ዐንዳንድ ጊዜ
		[_]3.Often (more than ten times the past four weeks)	[_]3.Seeda	[_]3. ብዙ ጊዜ
907	**In the past four weeks, did you or any household member have to eat some foods that you really did not want to eat because of a lack of resources to obtain other types of food?**			
	Sau shoole lamalara hoongunni kainohunni ati woy miniki manni giddo mittu ita hasidhinayikki sagalaittineegentinooni	0. [_]No	0. [_]Dee'ni	0. [_]አይደለም
	ባለፉት ዐራት ሳምንታት መግዛት ካለመቻል የተነሳ ዐንቺ ወይም ከቤተሰብሽ ዐባላት መብላት የማትፈልጉትን ምግብ በልታችሁ ታውቃላችሁ	1. [_]Yes	1. [_]Eewa	1. [_]አዎን
908 Skip ዝለል	**How often did this happen in the past four weeks?**			
	Kuni mageeshshi geeshshaatisau shoole lamalara ikkeuohu	[_]1.Rarely (once or twice in the pastfour weeks)	[_]1.Harancho hanna	[_]1.ጥቂት ጊዜ
	ባለፉት 4 ሳምንታት ለምን ያህል ጊዜ ዐጋጠመሽ	[_]2.Sometimes (three to ten times inthe past four weeks)	[_]2.Sae sae	[_]2. ዐንዳንድ ጊዜ
		[_]3.Often (more than ten times the past four weeks)	[_]3.Seeda	[_]3. ብዙ ጊዜ
	**In the past four weeks, did you or any household member have to eat a smaller meal than you felt youneeded because there was not enough food?**			
909	Sau shoole lamalara atway minikki maate giddomitto ikkado sagala heera hoogatenni kainohunni shiima ikkado sagala sagalino	0. [_]No	0. [_]Dee'ni	0. [_]አይደለም

	ባለፉት ዐራት ሳምንታት መግዛት ካለመቻል የተነሳ ዐንቺ ወይም ከቤተሰብሽ ዐባላት መብላት ከሚገባው መጠን በታች በልታችሁ ታውቃላችሁ	1. [_]Yes	1. [_]Eewa	1. [_]አዎን
	**How often did this happen in the past four weeks?**			
910 Skip ዝለል	Sagalino sau shoole lamalara mageshshi yanna geeshshati xaadinohu	[_]1.Rarely (once or twice in the pastfour weeks)	[_]1.Harancho hanna	[_]1.ጥቂት ጊዜ
	ባለፉት 4 ሳምንታት ለምን ያህል ጊዜ ዐጋጠመሽ	[_]2.Sometimes (three to ten times inthe past four weeks)	[_]2.Sae sae	[_]2. ዐንዳንድ ጊዜ
		[_]3.Often (more than ten times the past four weeks)	[_]3.Seeda	[_]3. ብዙ ጊዜ
	**In the past four weeks, did you or any other household member have to eat fewer meals in a day because there was not enough food?**			
911	Sau shoole lamalara ikkado sagale heera hoogatenni kainohunni at woy minikkki maate giddo mittu barru giddo sagala noosi yanna sagalinokki yanna no	0. [_]No	0. [_]Dee'ni	0. [_]አይደለም
	ባለፉት 4 ሳምንታት በቂ ምግብ ካለመኖሩ የተነሳ ዐንቺ/ከቤተሰብሽ መካከል ምብላት ባለበት ሰዐት ያልተመገበ ዐለ	1. [_]Yes	1. [_]Eewa	1. [_]አዎን
912 Skip ዝለል	**How often did this happen in the past four weeks?**			
	Sau lamalara mee yanna geeshshaati xaadinonehu	[_]1.Rarely (once or twice in the pastfour weeks)	[_]1.Harancho hanna	[_]1.ጥቂት ጊዜ
	ባለፉት 4 ሳምንታት ለምን ያህል ጊዜ ዐጋጠመሽ	[_]2.Sometimes (three to ten times inthe past four weeks)	[_]2.Sae sae	[_]2.ዐንዳንድ ጊዜ
		[_]3.Often (more than ten times the past four weeks)	[_]3.Seeda	[_]3. ብዙ ጊዜ
	**In the past four weeks, was there ever no food to eat of any kind in your household because of lack of resources to get food?**			

913	Sau lamalara anjetennikainohunni sagale minigiddo hooge epentewo	0. [_]No	0. [_]Dee'ni	0. [_]አይደለም
	ባለፉት 4 ሳምንታት መግዛት ካለመቻል የተንሳ ማንኛዉም ዐይነት ምግብ ከቤትሽ ጠፍቶ ያውቃል	1. [_]Yes	1. [_]Eewa	1. [_]አዎን
	**How often did this happen in the past four weeks?**			
914	Ikkiromageshshi yanna geeshshaati	[_]1.Rarely (once or twice in the pastfour weeks)	[_]1.Harancho hanna	[_]1.ጥቂት ጊዜ
ዝለል	ባለፉት 4 ሳምንታት ለምን ያህል ጊዜ ዐጋጠመሽ	[_]2.Sometimes (three to ten times inthe past four weeks)	[_]2.Sae sae	[_]2. ዐንዳንድ ጊዜ
		[_]3.Often (more than ten times the past four weeks)	[_]3.Seeda	[_]3. ብዙ ጊዜ
915	**In the past four weeks, did you or any household member go to sleep at night hungry because there was not enough food?**			
	Sau shoole lamalara ati woy minnikki maate giddo mittu sagalete anjoninni kainohunni hudiisannassi gaxinohu no	0. [_]No	0. [_]Dee'ni	0. [_]አይደለም
	ባለፉት 4 ሳምንታት ዐንቺ/ከቤተሰብሽ መካከል ከምግብ ዕጥረት የተነሳ ዕየራበው ሳይበላ የተኛ ዐለ	1. [_]Yes	1. [_]Eewa	1. [_]አዎን
916 Skip ዝለል	**How often did this happen in the past four weeks?**			
	Ikkiro mageeshshi yanna geeshshaati sau shoole lamalara	[_]1.Rarely (once or twice in the pastfour weeks)	[_]1.Harancho hanna	[_]1.ጥቂት ጊዜ
	ባለፉት 4 ሳምንታት ለምን ያህል ጊዜ ዐጋጠመሽ	[_]2.Sometimes (three to ten times inthe past four weeks)	[_]2.Sae sae	[_]2. ዐንዳንድ ጊዜ
		[_]3.Often (more than ten times the past four weeks)	[_]3.Seeda	[_]3. ብዙ ጊዜ
917	**In the past four weeks, did you or any household member go a whole day and night without eating anything because there was not enough food?**			

	Sau shoole lamalara at way minikki maate giddo mittu sagalete anjenni kainohunni sagalikkinni 24 saate keeshshinohu no	0. [_]No	0. [_]Dee'ni	0. [_]አይደለም
	ባለፉት 4 ሳምንታት ዐንቺ/ከቤተሰብሽ መካከል ከምግብ ዕጥረት የተነሳ ሳይመገብ 24 ሰዐት የቆየ ዐለ	1. [_]Yes	1. [_]Eewa	1. [_]አዎን
918 Skip ዝለል	**How often did this happen in the past four weeks?**			
	Ikkiro mageeshshi yanna geeshshaati kalaqaminohu	[_]1.Rarely (once or twice in the pastfour weeks)	[_]1.Harancho hanna	[_]1.ጥቂት ጊዜ
	ባለፉት 4 ሳምንታት ለምን ያህል ጊዜ ነው ያጋጠመሽ	[_]2.Sometimes (three to ten times inthe past four weeks)	[_]2.Sae sae	[_]2. ዐንዳንድ ጊዜ
		[_]3.Often (more than ten times the past four weeks)	[_]3.Seeda	[_]3. ብዙ ጊዜ
919	**Have you received any food support in the past month?**			
	Sai aganira ayita sagalete kaalo adhootta	0. [_]No	0. [_]Dee'ni	0. [_]አይደለም
	ባለፈው ወር ማንኛውም የምግብ ዕርዳታ ዐግኝተሻል	1. [_]Yes	1. [_]Eewa	1. [_]አዎን
920	**How do you cope at times when you are running out of food in the house?**			
	Sagala mini giddo heedhu kkinni gatturo maat assahahu	1[_].Reduce number of meals	1[_]Adhaw sagaleaanoheayeajishe ena	1[_]የሚወሰደውን የምግብ ድግግሞሽ ቀንሳለሁ
	በቤት ውስጥ የምግብ ዕጥረት ሲያጋጥምሽ ምን ዐማራጭ ነው የምትወስጂው	2[_].Reduce meal size	2[_]Sagalete geesha ajiplema	2[_]የምግብ መጠን ቀንሳለሁ
		3[_].Borrowing	3[_]Liqiirema	3[_]ዕበደራለሁ
		4[_].Petty trade	4[_]Hirary sagale hasileme	4[_]የሚሸጥ ነገር ፈልጋለሁ
		5[_].Consume stored food (seed)	5[_]Gootam giddo nosagale harunsitmo	5[_]በጎተራ ያለ ምግብ ዕጠቀማለሁ
		6[_].Migration for labour	6[_]Loosoho walekachcha horeema	6[_]ለስራ ወደ ሌላ ዐካባቢ ሔዳለሁ

		7[_].Sell of farm tools	7[_]Hwurayi udene hirana	7[_]የማረሻ ዕቃዎችን ሽጣለሁ
		8[_].Sale charcoal/fire wood	8[_]Ishine hirema	8[_]ቆሻሻ ሽጣለሁ
		9[_].Daily labor	9[_]Barulooso losema	9[_]የቀን ሥራ ሰራለሁ
		10[_].Safety Net	10[_]Kaalo hasireema	10[_]ዕርዳታ ዐገኛለሁ
		11[_].Sell of farm animals	11[_]Ishine adhe itema	11[_]ከቆሻሻ ወስጄ በላለሁ
		12[_].Other(specify)	12[_]Welu noose keeli	12[_]ሌላ ካለ ጥቀስ/ሽ

Section 10: Household dietary diversity
Kiffile 10. Mini mate sagalete danisagala
ክፍል 10. የቤተሰቡ የምግብ ዐመጋገብ
Now I would like to ask you about the types of foods that you or anyone else in your household ate yesterday during the day and at night either separately or combined with other foods.
ቤተሰቡ በ 24 ሰዐት ዉስጥ የተመገበውን የምግብ ዐይነት የሚገለፅ መጠይቅ (ቤተሰቡ ቁርስ፣ምሳ፣ዕራት የሰራውን ወይም የተመገበውን ምግብ ጠይቅ የሚስማማውን ቦታ ዐመልክት)

1000	**Were there any foods that were not prepared for the in the house because it was a fasting day?**			
	Qatume ikkewo daafira loonsoyikki sagale no	0. [_]No	0. [_]Dee'ni	0. [_]አይደለም
	ጾም ስለሆነ ያልተሰራ ምግብ ዐለ	1. [_]Yes	1. [_]Eewa	1. [_]አዎን
1001	**Could you tell me the types of foods that were prepared in the house and that you or anyone else in your household ate?**			
	Loonsoyi sagale kulatoe	[_]Breakfast		[_]ቁርስ
	ለቤተሰቡ የተሰራውን የምግብ ጊዜ ትነግሪኛለሽ	[_]Lunch		[_]ምሳ
		[_]Dinner		[_]ዕራት
		[_]Others		[_]ሌላካለ ጥቀስ/ሽ

1002	**Any bread, rice, pasta, biscuits, or any other foods made from millet, sorghum, maize, rice, wheat?**			
	Daabbo,paarta,ruuze,koshoro raino sagale woy ajjunni ,badalatenni ,hayixunni,qamadetenni,bashanqunniy loonsoonni sagale woy wolu quminni,xawisi	0. [_]No	0. [_]Dee'ni	0. [_]አይደለም
	ዳቦ፤ ፓስታ፤ ሩዝ፤ ብስኩት፤ ኩኪስ፤ ወይም ማንኛውም ነገር ከአጃ ከበቆሎ ገብስ፤ ሰንዴ፤ ማሽለ፤ወይም ሌላ አህል ዘር የተሰራ	1. [_]Yes	1. [_]Eewa	1. [_]አዎን
1003	**Any potatoes, bulla, kocho or any other food made from roots or tubers?**			
	Maxaaxeesla, diinicha, boyina,lawinore saga'lino?	0. [_]No	0. [_]Dee'ni	0. [_]አይደለም
	ማንኛውም ነጭ ድንች፤ ቦይና፤ እንሰት (ማንኛውም ነጭ ስራስር)	1. [_]Yes	1. [_]Eewa	1. [_]አዎን
1004	**Any vegetables?**			
	Wole aye ataakilte saga'lino?	0. [_]No	0. [_]Dee'ni	0. [_]አይደለም
	ማንኛውም አታክልት	1. [_]Yes	1. [_]Eewa	1. [_]አዎን
1005	**Any fruits?**			
	Wole aye gumma	0. [_]No	0. [_]Dee'ni	0. [_]አይደለም
	ማንኛውም ፍራፍሬ	1. [_]Yes	1. [_]Eewa	1. [_]አዎን
1006	**Any beef, pork, lamb, goat, rabbit wild game, chicken, duck, or other birds, liver, kidney, heart, or other organ meats?**			
	Bootu,mancheemete,gereewo,mellenna hilleessa,Lukko,daakiyye, Afale,mule,wodana,woy wole godowu giddo malla?	0. [_]No	0. [_]Dee'ni	0. [_]አይደለም
	የበሬ፤ የበግ/የፍየል የአሳማ,የጥንቸል ,ዶሮ፤ እርግብ/ወፍ (ጉበት፤ ኩላሊት፤ የልብ ወይም ማንኛውም የውስጥ ሰውነት ክፍል)	1. [_]Yes	1. [_]Eewa	1. [_]አዎን
1007	**Any eggs?**			
	Ayee quuphe	0. [_]No	0. [_]Dee'ni	0. [_]አይደለም
	ማንኛውም እንቁላል	1. [_]Yes	1. [_]Eewa	1. [_]አዎን
1008	**Any fresh or dried fish or shellfish?**			
	Ayee qilxi'me	0. [_]No	0. [_]Dee'ni	0. [_]አይደለም
	ማንኛውም አሳና የአሳምርት	1. [_]Yes	1. [_]Eewa	1. [_]አዎን
1009	**Any foods made from beans, peas, lentils, or nuts?**			

	Aye segale baqeluuni, atarunni ,qibaatete qumma shumburunni qixxeessinoonni sagale saga’lino?	0. [_]No	0. [_]Dee’ni	0. [_]አይደለም
	ማንኛውም ምግብ ከ ባቄላ፤አተር፤ ምሰር ወይም ሌላ ጥራጥሬ	1. [_]Yes	1. [_]Eewa	1. [_]አዎን
1010	**Any cheese, yogurt, milk or other milk products?**			
	Ayibe/geinto saga’lino?	0. [_]No	0. [_]Dee’ni	0. [_]አይደለም
	ማንኛውም አይብ፣እርጎ፣ወተት ወይም ሌላ የወተት ምርቶች	1. [_]Yes	1. [_]Eewa	1. [_]አዎን
1011	**Any foods made with oil, fat, or butter?**			
	Zayitetenni woy buurumi loonsoonni sagala	0. [_]No	0. [_]Dee’ni	0. [_]አይደለም
	በዘይት ወይም በቅቤ የተሰራ ምግብ	1. [_]Yes	1. [_]Eewa	1. [_]አዎን
1012	**Any sugar or honey?**	0. [_]No	0. [_]Dee’ni	0. [_]አይደለም
	ማንኛውም ስኩዋርና ማር	1. [_]Yes	1. [_]Eewa	1. [_]አዎን
1013	**Any other foods, such as condiments, coffee, tea?**	0. [_]No	0. [_]Dee’ni	0. [_]አይደለም
	ቡናና ሻይ	1. [_]Yes	1. [_]Eewa	1. [_]አዎን
1014	**Alchol drink**			
	Diribisanno ago (Birra, Xesiixella,Areqe	0. [_]No	0. [_]Dee’ni	0. [_]አይደለም
	ዐልኮል መጠጦች (ቢራ፣ ዐረቄ፣ጠጅ፣ጠላ)	1. [_]Yes	1. [_]Eewa	1. [_]አዎን
1015	**Others specify**			
	Welere			
	ሌላ ካለ ዘርዝር			
